# Whole Blood DNA Aberrant Methylation in Pancreatic Adenocarcinoma Shows Association with the Course of the Disease: A Pilot Study

**DOI:** 10.1371/journal.pone.0037509

**Published:** 2012-05-22

**Authors:** Albertas Dauksa, Antanas Gulbinas, Giedrius Barauskas, Juozas Pundzius, Johannes Oldenburg, Osman El-Maarri

**Affiliations:** 1 Institute for Digestive Research, Lithuanian University of Health Sciences, Kaunas, Lithuania; 2 Department of Surgery, Lithuanian University of Health Sciences, Kaunas, Lithuania; 3 Institute of Experimental Hematology and Transfusion Medicine, University of Bonn, Germany; The University of Kansas Medical Center, United States of America

## Abstract

Pancreatic tumors are usually diagnosed at an advanced stage in the progression of the disease, thus reducing the survival chances of the patients. Non-invasive early detection would greatly enhance therapy and survival rates. Toward this aim, we investigated in a pilot study the power of methylation changes in whole blood as predictive markers for the detection of pancreatic tumors. We investigated methylation levels at selected CpG sites in the CpG rich regions at the promoter regions of p16, RARbeta, TNFRSF10C, APC, ACIN1, DAPK1, 3OST2, BCL2 and CD44 in the blood of 30 pancreatic tumor patients and in the blood of 49 matching controls. In addition, we studied LINE-1 and Alu repeats using degenerate amplification approach as a surrogate marker for genome-wide methylation. The site-specific methylation measurements at selected CpG sites were done by the SIRPH method. Our results show that in the patient’s blood, tumor suppressor genes were slightly but significantly higher methylated at several CpG sites, while repeats were slightly less methylated compared to control blood. This was found to be significantly associated with higher risk for pancreatic ductal adenocarcinoma. Additionally, high methylation levels at TNFRSCF10C were associated with positive perineural spread of tumor cells, while higher methylation levels of TNFRSF10C and ACIN1 were significantly associated with shorter survival. This pilot study shows that methylation changes in blood could provide a promising method for early detection of pancreatic tumors. However, larger studies must be carried out to explore the clinical usefulness of a whole blood methylation based test for non-invasive early detection of pancreatic tumors.

## Introduction

Pancreatic cancer is the fourth most common cause of cancer*-*related mortality worldwide, with the majority of cases leading to death within a relatively short time [1]. Pancreatic cancer is associated with no or minimal symptoms making detection of this potentially curable malignancy difficult to achieve. Hence, the late onset of symptoms and delayed diagnosis of the disease severely limits survival in these patients. Currently, the overall five year survival rate following pancreaticoduodenectomy for pancreatic cancer is as low as 15–20% [2]. An early, reliable, and noninvasive test with the potential to increase the rates of detection and to provide early warning for the presence of the malignancy is urgently required. Such a test would increase the rate of survival and the cure rate. However, knowledge on the molecular pathogenesis of pancreatic cancer and availability of possible biomarkers with clinical value to be used for the development of such a test are limited.

Most investigations of biomarkers in patients with cancers have focused on the assessment of differences in methylation levels between tumor and histologically normal tissue. Indeed, aberrant DNA methylation has been found to play an important role in the development, progression and outcome of most cancers [3]. Two forms of frequently observed methylation changes in cancer take place: global hypomethylation that is largely observed in gene-poor regions and repetitive DNA and gene-specific hypermethylation of CpG islands [4]. Hypermethylation of promoter regions has been frequently described in pancreatic [5]–[8] and other cancers [9]–[11].

However, since most studies are focusing on methylation changes in the tumor tissue itself, in such cases, access to the diseased tissue by surgery is required, which limits its clinical utility as non-invasive early diagnosis. Alternatively, body fluids such as saliva [12], sputum [13], bronchoalveolar lavage [14], urine [15] and stools [16] that are in contact with tumors are potential sources of cancer cell DNA for analysis of epigenetic changes. Another readily available biological sample that can be obtained noninvasively is peripheral blood. It is well known that DNA fragments are frequently abundant in sera/plasma of cancer patients (about 200 ng/ml) [17] with significantly higher levels in patients with metastasis [18]. A number of studies have evaluated the potential of circulating tumor-related DNA in serum for molecular diagnosis and prognosis of various types of cancer [19], [20].

Also, most importantly, there is growing evidence that methylation changes arise systematically and may be measured in surrogate tissue, such as peripheral blood leukocytes. Most of the studies on quantitative methylation in whole blood to differentiate tumors patients from healthy controls have used global methylation assays targeting repetitive elements (like Alu and LINE1), while only a few have investigated specific loci. In pancreatic tumor patients, only one previous report has been published investigating the power of DNA methylation changes in leukocytes to discriminate pancreatic cancer patients from healthy controls [21]. The authors could identify five sites (IL10, LCN2, ZAP70, AIM2, TAL1) that identify pancreatic cancer patients from controls. However, they did not study repetitive elements and did not correlate the methylation values to the clinical parameters or to the progression of the disease.

The aim of the present pilot study was to use a highly sensitive quantitative method to determine whether a difference exists between selected tumor suppressor genes (p16, RARbeta, TNFRSF10C, APC, ACIN1, DAPK1, 3OST2, BCL2 and CD44) and genome-wide repetitive sequence (LINE-1 and Alu) methylation in peripheral blood of subjects with pancreatic cancer and healthy controls. Moreover, we correlated the methylation values with clinical-morphological variables and determined the influence on survival rates. Additionally, we examined the methylation level in corresponding malignant tissue samples. Our pilot study shows that DNA methylation changes in whole blood could indeed be associated with the presence of pancreatic tumors and with the progression of the disease.

## Results

### Peripheral Blood Derived DNA Methylation Analysis

In this pilot study, we investigated locus specific methylation at selected CpGs at Alu and LINE-1 repeats and at promoter regions of nine tumor suppressor genes. Using the SIRPH protocol (SNuPE combined with ion pair reverse phase HPLC) [22], [23] we measured methylation at three CpG sites, in the Alu consensus sequence, while two CpG sites were analyzed at LINE-1 repeats. At tumor suppressor genes we measured one CpG site in the p16, BCL2, DAPK1, TNFRSF10C, CD44 promoter regions, while two CpG sites were measured in the APC, 3OST2, ACIN1 and RARbeta promoter regions. Methylation levels at selected CpG sites were quantitatively evaluated by bisulfite treatment of the DNA and subsequent quantification of methylation at selected specific CpG sites by the SIRPH protocol. Details of the studied regions are shown in Figure S1. Blood samples for DNA extraction were available from 26 subjects of the 30 pancreatic cancer cases. The clinical characteristics of the patients included in this study are summarized in Table 1. Controls used in this study were matching for age and gender distribution.

**Table 1:** Clinical characteristics of patients included in this study.

Characteristic		Cases (%)	Control (%)
Age (years)	<65	12 (40.0)	30 (61.2)
	≥65	18 (60.0)	19 (38.8)
Gender	female	16 (53.3)	30 (61.2)
	male	14 (46.7)	19 (38.8)
Stage (UICC classification)	I/II	8 (26.6)	
	III/IV	22 (72.4)	
T stage (TNM classification)	T1/T2	3 (10.0)	
	T3/T4	27 (90.0)	
Lymph node involvement	negative	7 (23.3)	
	positive	23 (76.7)	
Invasion into blood vessels	negative	14 (46.7)	
	positive	16 (53.3)	
Invasion into lymph vessels	negative	7 (23.3)	
	positive	23 (76.7)	
Perineural spread	negative	8 (26.7)	
	positive	22 (73.3)	
Cell differentiation grade	G1/G2	4 (13.3)	
	G3/G4	20 (66.7)	
Invasion into peripancreatic tissue	negative	8 (26.7)	
	positive	22 (73.3)	

**Figure 1:**
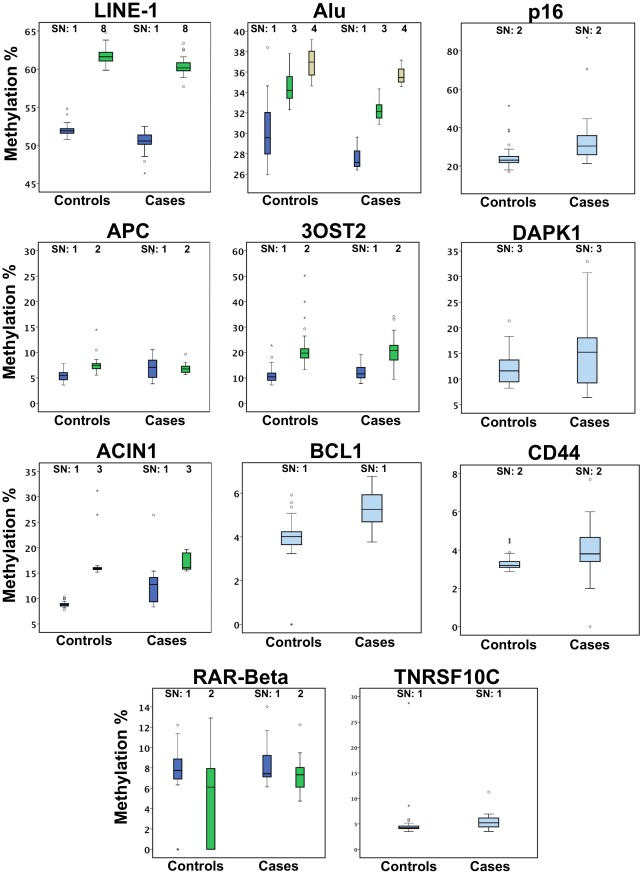
Distribution of various CpGs relative methylation levels in peripheral blood. Boxes extend from 25th to 75th percentiles and are divided by a solid line representing the median of each group. Whiskers extend from 5th to 95th percentiles. Each outlier is denoted by a dot.

The average methylation levels at each CpG site for blood derived DNA from cancer patients and controls are summarized in Table 2 and Figure 1. We could observe two trends. First, at Alu and LINE-1 repeats, mean methylation level in patients was slightly lower than mean methylation level in controls. Second, at tumor suppressor genes mean methylation level was higher in patients than in controls, with the exception of APC SN2 (Figure 1). Methylation level difference was highly significant (p<0.0001) for all CpGs in LINE-1, Alu repeats and in p16, APC SN1, ACIN1 SN1 and SN3, BCL2, CD44, TNFRSF10C.

**Table 2:** Peripheral blood derived DNA mean methylation comparison in cases and controls (Mann-Whitney test).

		Control		Cases		
	Primer	Mean	SD	Mean	SD	*P* values
***LINE-1***	SN-1	51.99	0.70	50.50	1.32	**.0000**
	SN-8	61.65	0.98	60.38	1.21	**.0000**
***Alu***	SN-1	30.02	2.57	27.52	0.93	**.0000**
	SN-3	34.53	1.41	32.21	0.95	**.0000**
	SN-4	36.95	1.30	35.65	0.73	**.0000**
***P16***	SN-2	24.24	5.66	33.92	14.58	**.0000**
***APC***	SN-1	5.35	0.96	7.91	4.88	**.0007**
	SN-2	7.47	1.39	6.86	0.93	**.0228**
***30ST2***	SN-1	10.96	2.86	12.24	3.08	.0513
	SN-2	21.00	6.30	20.75	5.69	.8586
***DAPK1***	SN-3	11.93	3.01	15.38	6.96	.0584
***ACIN1***	SN-1	8.85	0.50	12.44	3.72	**.0000**
	SN-3	16.39	2.68	17.19	1.64	**.0127**
***BCL2***	SN-1	3.66	1.45	5.30	.83	**.0000**
***CD44***	SN-2	3.33	0.41	3.99	1.39	**.0000**
***RARBeta***	SN-1	7.59	4.49	8.14	1.77	.8760
	SN-2	5.38	4.09	7.28	1.61	.0688
***TNFRSF10C***	SN-1	4.92	3.59	5.39	1.50	**.0000**

SD- standard deviation.

### Peripheral Blood Relative Methylation Association with Presence of Pancreatic Ductal Adenocarcinoma

After observing significant differences in blood methylation between patients and healthy controls, we performed logistic regression analysis to examine the association between relative methylation levels at each of the investigated regions (gene promoter regions and repetitive elements) and pancreatic ductal adenocarcinoma presence in patients (Table 3). In this model, methylation was categorized into three groups divided at the 33rd and 66th percentiles, with the highest tertile of relative methylation serving as reference for Alu and LINE-1. The lowest tertile served as reference for promoters of tumor suppressor genes p16, APC, 30ST2, DAPK1, ACIN1, BCL2, CD44, RARbeta, TNFRSCF10C. We observed that in patients with lowest tertile of LINE-1 SN1 (OR 10.4, 95%CI 2.3-47.7) and LINE-1 SN8 (OR 7.8, 95%CI 1.9-32.2), relative methylation had a significantly higher prevalence of pancreatic cancer. Similar findings were observed in the lowest tertile of Alu SN1 (OR 8.2, 95%CI 1.9-35.6), Alu SN3 (OR 7.4, 95%CI 1.9-29.4) and Alu SN4 (OR 7.7, 95%CI 1.8-32.3) (Table 3).

**Table 3:** Blood-derived DNA relative methylation is associated with the presence of pancreatic ductal adenocarcinoma in patients.

Gene loci	Site	Relative methylation levels (tertiles in controls)	Controls (%)	Pancreatic adenocarcinoma patients (%)	Adjusted OR* (95% CI)
***LINE-1***	SN1	High	16 (32.7)	1 (3.3)	1.0 (reference)
		Middle	17 (34.7)	1 (3.3)	0.3 (0.3–3.1)
		Low	16 (32.7)	24 (80)	**10.4 (2.3–47.7)**
	SN8	High	14 (28.6)	3 (10)	1.0 (reference)
		Middle	18 (36.7)	2 (6.7)	0.5(0.1–3.5)
		Low	16 (32.7)	21 (70)	**7.8(1.9**–**32.2)**
***Alu***	SN1	High	16 (32.7)	0 (0)	1.0 (reference)
		Middle	17 (34.7)	5 (16.7)	1.8(0.4–9.3)
		Low	16 (32.7)	21 (70)	**8.2(1.9**–**35.6)**
	SN3	High	16 (32.7)	0 (0)	1.0 (reference)
		Middle	17 (34.7)	2 (6.7)	0.6(0.1–4.3)
		Low	16 (32.7)	24 (80)	**7.4(1.9**–**29.4)**
	SN4	High	16 (32.7)	0 (0)	1.0 (reference)
		Middle	17 (34.7)	5 (16.7)	1.7(0.3–8.1)
		Low	16 (32.7)	21 (70)	**7.7(1.8–32.3)**
***P16***	SN-2	Low	16 (32.7)	2 (6.7)	1.0 (reference)
		Middle	17 (34.7)	3 (10.0)	1.0(0.1–7.4)
		High	16 (32.7)	21 (70.0)	**10.4(2.0–54.7)**
***APC***	SN-1	Low	16 (32.7)	5 (16.7)	1.0 (reference)
		Middle	17 (34.7)	4 (13.3)	0.9(0.2–4.0)
		High	16 (32.7)	17 (56.7)	**3.7(1.1–12.8)**
	SN-2	Low	15 (30.6)	14 (46.7)	1.0 (reference)
		Middle	17 (34.7)	5 (16.7)	4.4(1.0–19.0)
		High	16 (32.7)	3 (10.0)	1.5(0.3–7.3)
***30st2***	SN-1	Low	16 (32.7)	5 (16.7)	1.0 (reference)
		Middle	17 (34.7)	6 (20.0)	0.8(0.2–2.7)
		High	16 (32.7)	15 (50.0)	2.1(0.7–6.8)
	SN-2	Low	16 (32.7)	10 (33.3)	1.0 (reference)
		Middle	17 (34.7)	5 (16.7)	0.4(0.1–1.3)
		High	16 (32.7)	11 (36.7)	0.9(0.3–2.6)
***DAPK-1***	SN-3	Low	16 (32.7)	7 (23.3)	1.0 (reference)
		Middle	17 (34.7)	4 (13.3)	0.4(0.1–1.5)
		High	16 (32.7)	15 (50.0)	1.6(0.6–4.6)
***ACIN1***	SN-1	Low	16 (32.7)	3 (10.0)	1.0 (reference)
		Middle	16 (32.7)	3 (10.0)	0.6(0.1–2.7)
		High	16 (32.7)	20 (66.7)	**4.2(1.2–14.8)**
	SN-3	Low	16 (32.7)	4 (13.3)	1.0 (reference)
		Middle	17 (34.7)	8 (26.7)	1.4(0.4–4.9)
		High	16 (32.7)	14 (46.7)	2.6(0.7–9.1)
***BCL2***	SN-1	Low	14 (28.6)	1 (3.3)	1.0 (reference)
		Middle	15 (30.6)	1 (3.3)	1.1(0.1–20.8)
		High	14 (28.6)	24 (80.0)	**33.8(3.6–314.3)**
***CD44***	SN-2	Low	16 (32.7)	2 (6.7)	1.0 (reference)
		Middle	17 (34.7)	4 (13.3)	2.1(0.3–13.2)
		High	16 (32.7)	20 (66.7)	**10.3(2.0–52.3)**
***RARBeta***	SN-1	Low	16 (32.7)	12 (40.0)	1.0 (reference)
		Middle	17 (34.7)	4 (13.3)	0.3(0.1–1.0)
		High	16 (32.7)	10 (33.3)	0.7(0.3–2.0)
	SN-2	Low	16 (32.7)	1 (3.3)	1.0 (reference)
		Middle	16 (32.7)	12 (40.0)	**12.1(1.4–106.4)**
		High	16 (32.7)	13 (43.3)	**14.2(1.6–125.1)**
***TNFRSF10C***	SN-1	Low	16 (32.7)	2 (6.7)	1.0 (reference)
		Middle	16 (32.7)	5 (16.7)	2.3(0.4–14.4)
		High	16 (32.7)	19 (63.3)	**11.8(2.2–64.1)**

* Model is adjusted for age groups (<65 years and> = 65 years).

The highest relative methylation tertile of seven tumor suppressor gene promoter regions also showed an association with the increased prevalence of pancreatic cancer. Single investigated CpG of p16 (OR 10.4, 95%CI 2.0-54.7), BCL2 (OR 33.8, 95%CI 3.6-314.3), CD44 (OR 10.3, 95%CI 2.0-52.3) and TNFRSF10C (OR 11.8, 95%CI 2.2-64.1) were associated with pancreatic cancer. However, DAPK1 (OR 1.6, 95%CI 0.6-4.6) does not affect the risk, as methylation levels were not significantly different between patients and controls. In contrast to repetitive elements where all studied CpGs showed the same significant results, when we analyzed more than one CpG in the tumor suppressor gene promoter region only one of the two CpGs reached a significant increase of relative risk in the highest tertile. This is true at APC SN1 (OR 3.7, 95% CI 1.1-12.8), ACIN1 SN1 (OR 4.2, 95% CI 1.2-14.8) and RARbeta SN2 (OR 12.1, 95% CI 1.4-106.4) promoter regions. These findings possibly indicate that not all CpG sites are equal in cancer risk prediction and larger number of CpGs covering larger areas of the CpG island must be analyzed to provide higher predictive values. As the methylation levels at the 3OST2 locus did not differ between cancer patients and controls no higher prevalence was linked to this locus.

### Association of Peripheral Blood Methylation with Clinical-morphological Features and Survival

Next, we searched for relationships between clinical-morphological features (Table 1) and Alu, LINE-1 hypomethylation or hypermethylation of the studied CpG loci of tumor suppressor genes. For this analysis, we grouped the highest and the middle tertiles for repetitive elements and the lowest and the middle tertile for CpG island loci into one variable. Further analysis was performed using 2×2 contingency tables (data not shown). Only high level of TNFRSF10C SN1 methylation was significantly associated with a positive perineural spread of cancer cells (OR 0.088, 95%CI 0.011-0.717).

**Figure 2:**
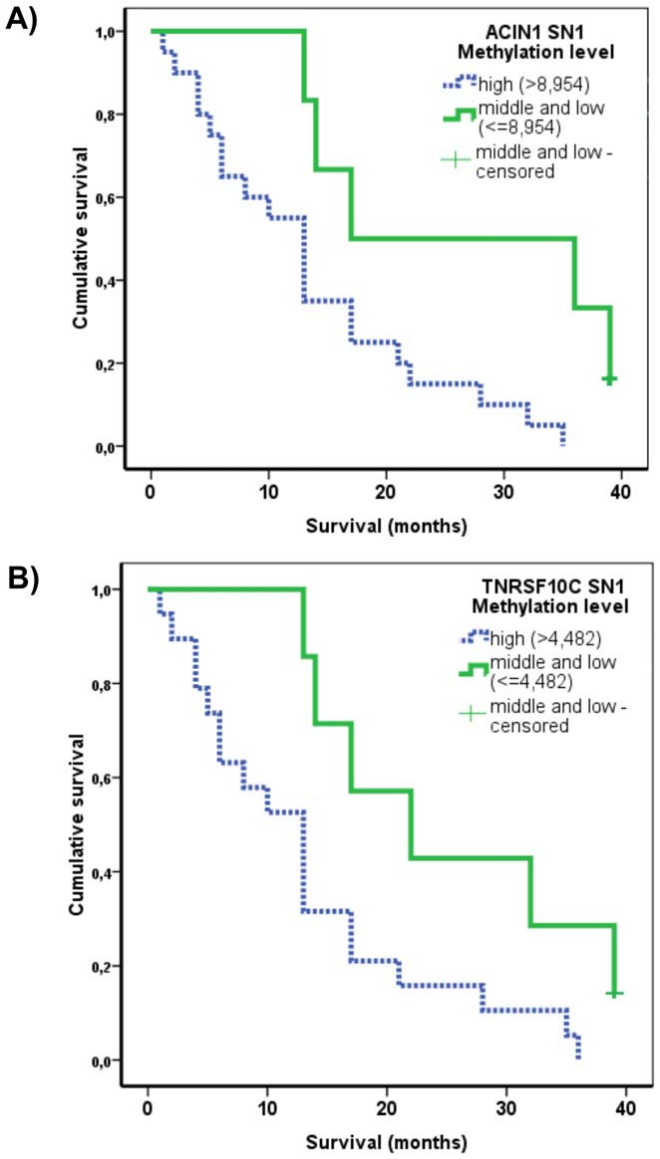
Kaplan-Meier survival estimates of overall survival for methylation levels in peripheral blood of cancer patients. ACIN1 SN1 (A), log rank = 0.012 and TNFRSF10C SN1 (B), log-rank = 0.023.

Further possible association of site-specific hypermethylation (at individual CpG sites) with cancer specific overall survival was evaluated using the Kaplan-Meier method and log-rank test. Hypomethylation of LINE-1 and Alu were not included in survival analysis as most of the samples (24/26) were in the lowest tertile. In this case, association of their hypomethylation with pancreatic cancer risk is already obvious. Median of the overall survival in pancreatic ductal adenocarcinoma patients was 13 months (95% CI 9.4-16.6). Association between hypermethylation of CpGs of promoters of tumor suppressor genes and cancer specific survival was evident only at two CpGs, namely TNFRSF10C SN1 and ACIN1 SN1. Regarding survival, the highest tertile of methylation of TNFRSF10C SN1 was significantly associated with shorter survival compared to the middle and the lowest tertile group (median survival 13 months vs. 22 months, log rank = 0.023) (Figure 2A). The highest tertile of methylation of ACIN1 SN1 was also significantly associated with shorter survival compared to the middle and the lowest tertile group (13 months vs. 17 months, log rank = 0.012) (Figure 2A).

### Comparison of Peripheral Blood and Cancer Tissue DNA Methylation

Mean methylation levels in peripheral blood were compared to corresponding pancreatic ductal adenocarcinoma tissues. Methylation levels of all investigated CpGs for Alu and LINE-1 were lower in cancer tissues compared to peripheral blood of cancer patients. Three groups of different methylation patterns could be distinguished in tissues. The first group of genes displayed higher methylation levels in cancer tissues than in total blood DNA (p16, APC, 3OST2, DAPK1). In the second group, methylation levels were approximately equal (BCL2, CD44, RARbeta, TNFRSF10C), while in the third group, methylation levels were lower (ACIN1). However, because of the lack of healthy ductal pancreatic tissue as control we cannot assess whether methylation levels in tumor tissue are specific for carcinoma or whether they merely reflect a tissue-specific variation between blood and pancreas.

### Inter-loci Correlation

Next, we investigated the interaction between different loci in terms of DNA methylation changes, i.e. inter loci correlations. For this purpose, we calculated the correlation of methylation between all loci pair wise and in all three studied tissue samples (blood samples from control group and from cancer patients).

**Figure 3:**
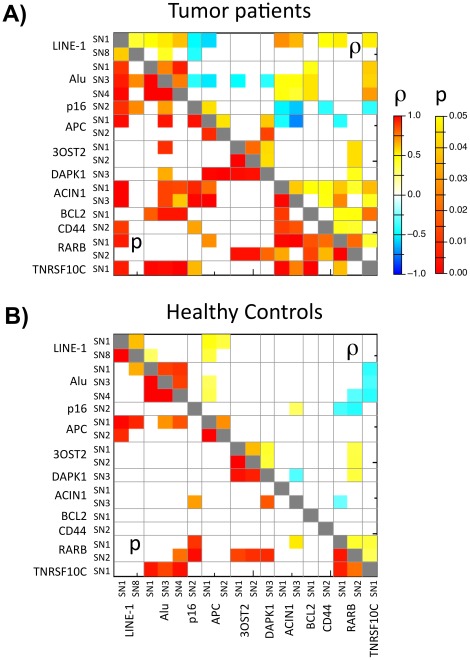
Heatmap showing p values (lower left triangle) and significant Spearman correlation coefficient (ρ: upper right triangle) between methylation levels at different CpGs in peripheral blood of pancreatic cancer patients (A) and control group (B) (p values of >0.05 are in white, the shown ρ-values are corresponding to p values <0.05).

We examined whether a correlation exists between hypermethylation levels of tumor suppressor genes and hypomethylation levels of repetitive elements in peripheral blood of pancreatic cancer patients. Using methylation levels as a continuous variable we found significant associations between methylation levels of different CpGs at promoter regions and repetitive elements (Figure 3). We could distinguish negative as well as positive correlations with the methylation levels of repeats. At least one CpG in each of p16, APC and 3OST2 showed negative correlation with methylation levels in CpG at Alu or LINE-1, while methylation levels in at least one CpG in each of DAPK1, ACIN1, BCL2, CD44, RARbeta and TNFRSF10C were positively correlated with methylation levels of one CpG site in the repeats. Likewise, inter-loci correlations between the non-repetitive loci showed both positive and negative correlations, with negative correlations between p16 and APC on the one hand, and ACIN1, CD44, RARbeta and TNFRSF10C on the other hand. Within the repeats themselves only positive correlations were observed.

In the blood of healthy controls, the inter-loci correlations as seen in the blood of cancer patients were largely maintained. However, the number of significant inter-loci correlations were mostly reduced (63 correlations in the blood of pancreatic cancer patients vs. only 29 correlations in the blood of healthy controls; Figure 3). This could be due to two reasons: 1) Lower variability of methylation levels in healthy controls compared to those found in the blood of cancer patients, which would result in less significant correlations. 2) Higher variability of methylation observed in tumor blood possibly caused by the presence of the tumor condition in the patients. This could reflect an organized body response against the challenge imposed on the body by the presence of tumor growth. What is of particular interest is the opposite direction of several correlations in the blood of healthy controls and the blood of cancer patients. Thus, correlations between APC SN1 and Alu SN3 and LINE-1 SN1 are positive in the blood of healthy controls, but negative in the blood of cancer patients. Moreover, correlations between TNFRSF10C SN1 and Alu SN1, 2 and 3 are negative in the blood of healthy controls but positive in the blood samples of cancer patients.

### Correlation between Methylation in Peripheral Blood and Malignant Tissues of Pancreatic Ductal Adenocarcinoma Patients

**Figure 4:**
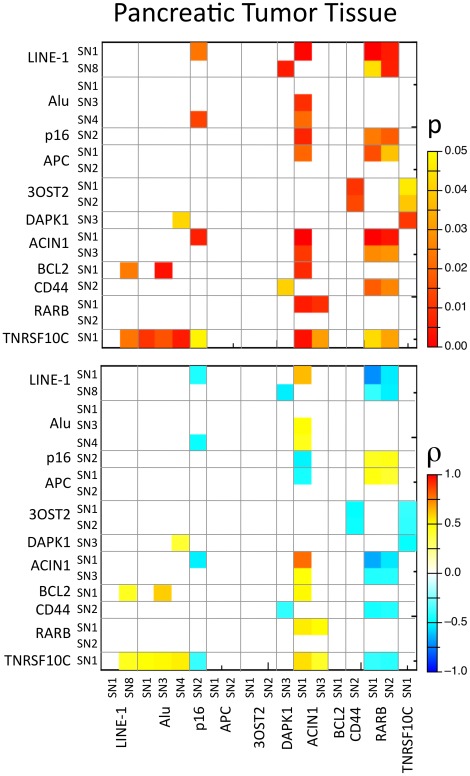
Heatmap showing p values and significant Spearman correlation coefficient (ρ) between methylation levels of different CpGs in paired peripheral blood and ductal adenocarcinoma tissues (p values of >0.05 are in white, the shown ρ-values are corresponding to p values <0.05).

The correlation between paired tumor and peripheral blood samples was estimated for each marker (Figure 4). In this context, three groups of correlations must be discussed: 1) correlation between the same loci in blood and in pancreatic tumor tissue, 2) reciprocal correlations, i.e. correlation between two loci with a correlation between blood and pancreatic tissue and between pancreatic tissue and blood, and 3) all other correlations. In the first category, we could find only one instance of ‘self’ correlating loci as methylation of ACIN1 SN1 exhibited a strong positive correlation between pancreatic cancer tissues and peripheral blood (r = 0.788, *p*<0.001). In the second category, we could distinguish two such correlations; first ACIN1 SN1 in blood correlated with p16 SN2 in pancreatic tumor tissue (ρ = −0.528, p = 0.006) and, reciprocally, p16 SN2 in blood correlated with ACIN1 SN1 in pancreatic tumor tissue (ρ = −0.519, p = 0.007). The second correlation in this category was found between RARB SN1 in blood and ACIN1 SN1 (ρ = 0.536, p = 0.005) and SN3 (ρ = 0.504, p = 0.009) in pancreatic tumor tissue and, reciprocally, ACIN1 SN1 (ρ = −0.664, p<0.001) and SN3 (ρ = −0.433, p = 0.027) in blood correlated with RARbeta SN1 in pancreatic tumor tissue.

Several correlations were observed in the third category. TNFRSF10C SN1 methylation in peripheral blood was well correlated with the methylation levels of LINE-1, Alu, p16, ACIN1 and RARbeta in pancreatic cancer tissue. Spearman’s negative correlation value ranged from −0.421 to −0.392 (*p* value 0.032–0.048), while the positive correlation value ranged from 0.423 to 0.56 (*p* value 0.005–0.048). At ACIN1 SN1, methylation levels in cancer tissues correlated well with methylation levels of LINE-1, Alu, p16 (see above), APC, BCL2, RARbeta and TNFRSF10C in peripheral blood. Spearman’s negative correlation value ranged from −0.519 to −0.455 (*p* value 0.007–0.02), while the positive correlation value ranged from 0.449 to 0.634 (*p* value 0.001–0.021). Moreover, RARbeta in blood correlated with TNFRSF10C, CD44, ACIN1, APC, p16 and LINE-1 in pancreatic tumor tissue. The negative correlation value ranged from −0.72 to −0.398 (*p* value<0.001–0.044), while the positive correlation value ranged from 0.44 to 0.47 (*p* value 0.015–0.038). These correlations are of great interest since TNFRSF10C SN1 as well as ACIN SN1 high methylation levels in peripheral blood were associated with poor survival.

## Discussion

Pancreatic tumors are often diagnosed in advanced stages of the disease when it has become too late for surgery to cure the disease. Therefore, one of the ultimate aims in clinical research is to develop a non-invasive early diagnostic test that would greatly enhance therapy and survival rates. To this end, we analyzed, in a pilot study, methylation status changes in the peripheral blood of pancreatic cancer patients and healthy controls. We observed lower levels of global methylation as measured by LINE-1 and Alu repetitive elements and higher methylation levels at promoters of tumor suppressor genes in the blood of pancreatic cancer patients compared to the blood of healthy controls. This was clearly associated with an increased risk for pancreatic cancer.

In the present pilot study, we found significant differences between cancer cases and controls, with p values for all of the five LINE-1 and Alu CpGs<0.0001. The methylation of Alu and LINE-1 repeats using degenerative amplification approaches was also previously shown to correlate with the 5-methylcytosine content in the human genome indicating that analysis of Alu and LINE-1 methylation may serve as a surrogate measure of genomic methylation levels [24]. Moreover, genomic DNA hypomethylation status in total blood DNA has recently been associated with higher risk for developing several cancers, such as colorectal, head and neck, bladder and breast cancers [25]–[28]. Therefore, hypomethylation of LINE-1 and Alu repeats from total blood could possibly correlate with the presence of inner organ tumors. This was found to be the case for LINE-1 in several studies [29]–[33], while other studies found no differences between cancer cases and controls [25], [34].

The association between methylation in the investigated genes in peripheral blood of pancreatic ductal adenocarcinoma and clinical-pathologic parameters in patients is important for the understanding of the pathogenesis of the disease. According to our results, methylation levels (or variations) of most of these genes and repetitive elements in peripheral blood had no significant correlation to clinical-pathologic parameters of patients. We did not find any association between global methylation levels (LINE-1 and Alu methylation) and tumor characteristics. This is consistent with other findings in colorectal adenoma and bladder cancer patients [27], [28]. However, higher methylation levels at TNFRSF10C promoter region in peripheral blood was positively associated with perineural tumor spread.

All of the above-described results demonstrate that levels of methylation at certain gene promoters in non-cancerous tissues (such as the studied blood) may be associated with survival in pancreatic ductal adenocarcinoma patients. We found that patients with high methylation levels of TNFRSF10C in peripheral blood DNA had shorter survival. Although it remains unclear whether TNFRSF10C plays a pro-apoptotic or an anti-apoptotic role in tumors, hypermethylation of TNFRSF10C has been reported in various human cancers [35]. One of the proposed mechanisms suggests a pro-apoptotic pathway, whereby the cancer cells gain advantage for proliferation and progression by inactivating TNFRSF10C through deletion or methylation of the promoter region [36], [37]. Our data are partly compatible with the latter of the proposed mechanisms since hypermethylation of TNFRSF10C in peripheral blood of pancreatic ductal adenocarcinoma patients is found to be associated with poor survival rates. Yet, the mechanism of TNFRSF10C downregulation, which is linked to the development of carcinogenic tissue, is not of direct relevance here as hypermethylation was observed in the blood surrogate tissue and not the tumor tissue itself. However, TNFRSF10C methylation in the blood of cancer patients positively correlated with LINE-1, Alu and ACIN1 and negatively with p16 and RARbeta in pancreatic tumor tissue (Figure 3). It is unlikely that higher methylation of LINE-1 or Alu or lower methylation of p16 and RARbeta in the cancer tissue itself are associated with tumor developments. Therefore, we postulate that hypermethylation of TNFRSF10C in blood exerts its effect on poor survival rates through its positive correlation with ACIN1 (SN1) tissue methylation, as the latter was also found to strongly correlate with methylation of blood ACIN1 (SN1) (Figure 3). We found high methylation levels at ACIN1 in peripheral blood DNA to be related to poor prognosis. Hypermethylation of the ACIN1 was also found in non-tumorous regions of early stage lung adenocarcinoma cases and this could be one of the early events in cancer development [38].

Methylation levels of the gene promoter regions differ between cancer DNA and peripheral blood DNA. We indentified three patterns of methylation in cancer tissues. Contrary to our expectations, some gene promoter regions in cancer tissues showed equal or lower methylation levels than those in blood DNA. Moreover, correlation between repetitive DNA sequences in some genes was negative, while in others, it was positive (Figure 3). These findings suggest that CpG island methylation gene promoter region and repetitive sequences are governed by different mechanisms and selective pressures of tumor progression, and that these two processes are possibly independent [39]. In a comprehensive study of satellite DNA hypomethylation, global DNA hypomethylation and hypermethylation of 55 gene loci in three types of ovarian epithelial tumors of different malignant potential and a variety of normal somatic tissues, it was found that DNA hypomethylation and hypermethylation usually co-exist in the same tumor but in different sequences [40]. The degree of gene hypermethylation was associated with the degree of malignancy of ovarian epithelial tumors. However, this association was independent of global DNA hypomethylation with the degree of malignancy. These results were consistent in hepatocellular carcinoma patients [41]. Clearly, these data show that some inter-relationship other than a mutual dependency exists between hypermethylation and hypomethylation of DNA. They possibly share some pathways in the poorly understood development resulting in these epigenetic changes. Also, different types of cancer-linked epigenetic abnormalities can interact in various ways [42]. Admittedly, conflicting data on dependence between global hypomethylation and targeted gene hypermethylation may be due to different methodologies and different CpG sites investigated in different studies.

Although this pilot study indicates a strong trend for a possible use of whole blood DNA methylation as marker for presence of pancreatic adenocarcinoma and its association with the progression of the disease, special care should be taken before such an approach is transferred to a clinical setup for the following reasons: 1) The relatively small patient sample size included in this pilot study; larger studies are needed to confirm informative markers in a majority of patients. 2) Lack of confirmation using an independent technology and a second independent set of patients. 3) Lack of verification of the markers specificity (i.e.: are the observed methylation changes specific for pancreatic adenocarcinoma) as the observed methylation changes could result from a general immune response to any tumor or any challenge/stress imposed on normal physiological conditions. Therefore, our data must be interpreted with care and over-interpretation must be avoided. Since this study is a pilot study, it does not provide ‘ready to use’ clinical markers (albeit clear trends) for early detection or prognosis of the disease. It is important here to mention that the measured methylation values in whole blood are nearly completely derived from nucleated leucocytes and the effect of circulating cancer cells or cell free DNA can be excluded. This is because the expected number of circulating cancer cells is negligible in comparison to the leukocytes (2 to 8 cells in 4.5 million leucocytes in a 7.5 ml whole blood) (Albertas please add reference and rearrange the references). In addition cell free DNA was discarded because only washed nuclei was used as input for the DNA extraction.

In summary, this pilot study shows that methylation of whole blood DNA is associated with pancreatic adenocarcinoma pathogenesis. These results indicate that combining multiple gene methylation profiles could provide greater accuracy than individual markers in predicting clinical outcomes. We support the growing evidence that methylation changes arise systematically and that they can be measured in surrogate tissues, such as peripheral blood. Therefore, use of peripheral leukocyte DNA methylation as a surrogate marker of gene silencing and genomic instability in target tumor tissue must be evaluated in larger studies.

## Materials and Methods

### Ethics Statement

This study was approved by the local Bioethics Committee of the Lithuanian University of Health Sciences (approval number BE-2-17) and written informed consent was obtained from all patients.

### Gene Selection

For this study, we selected genes of different metabolic pathways, as certain genes involved in cellular pathways, such as signal transduction, apoptosis, cell to cell communication, cell cycles and cytokine signaling, are down-regulated in cancers and may be considered potential tumor suppressor genes [43]. Therefore, we investigated, p16 [44] and APC [45], as they have been shown to be methylated at variable frequencies in pancreatic cancer [46], and apoptotic genes. ACIN1, DAPK1, TNFRSF10C, BCL2 [34], [38], [47]; 3OST2 [8], CD44 [48], and RARbeta [49] are found to be frequently methylated in other human cancers [34], [38], [47], [50]. For surrogate markers of global methylation, we selected long interspersed nucleotide elements (LINE-1) and the most abundant short interspersed nucleotides in the human genome (Alu).

### DNA Samples

This study included 30 pancreatic ductal adenocarcinoma patients diagnosed at the Department of Hepato-pancreato-biliary Surgery (Kaunas Medical University Hospital, Lithuania) between August 2005 and January 2007. All diagnoses were based on histopathological evidence and patients with adenocarcinoma, the most common histological type of pancreatic cancer, were included. The clinical-pathological characteristics of these pancreatic ductal adenocarcinoma patients based on the International Union against Cancer TNM classification are summarized in Table 1 [51]. Patients had neither undergone chemotherapy or radiotherapy prior to surgery, nor did they have any other diagnosed cancer. Blood samples from the patients were taken directly prior to surgery. The control population consisted of 49 patients with benign diseases (12 with inguinal hernias, eight with primary ventral hernias and 29 with cholelithiasis). Primary pancreatic tumors samples were surgically obtained from patients who underwent resection for pancreatic cancer. Samples were then divided into two portions. The first portion was fixed in formalin and was submitted for routine histopathological examination, while the second portion was immediately snap-frozen and stored in liquid nitrogen at −80°C until use in further experiments. Hematoxylin and eosin-stained slide from a frozen block was reviewed. The tumor cell cellularity was at least 80% in all samples.

### DNA Extraction

Five ml corresponding peripheral venous blood was available from twenty-six patients with pancreatic ductal adenocarcinoma. Forty-nine peripheral venous blood samples were obtained from control population. About 25 mg of pancreatic ductal adenocarcinoma tissue was taken for DNA extraction. Genomic DNA was extracted from tumor tissue and peripheral blood by DNeasy® Blood and Tissue Kit (Qiagen, Hilden, Germany) according to the manufacturer’s recommendation.

### Bisulfite Modification and PCR

200 ng of genomic DNA was subjected to bisulfite conversion with the EpiTect Bisulfite kit (Qiagen, Hilden, Germany) according to the manufacturer’s recommendation. Specific primers were designed with the assistance of the Methprimer software on http://www.urogene.org/methprimer (Table S1). PCR was performed in a 200 µl PCR tube and with a final volume of 30 µl, containing 6 pmol of each primer, 200 µmol/L of each dNTP, 1.5 U of HOT FIREPol DNA polymerase (Solis BioDyne, Tartu, Estonia) in buffer B containing 2.5 mmol/L MgCl2 and 2 µl of bisulfite-treated DNA as template. The initial denaturation (97°C, 15 minutes) was followed by 37 cycles of one minute at 95°C, one minute at primer specific temperatures (Table S1), one minute at 72°C, and a final extension step at 72°C for 10 minutes.

### Site-specific Methylation by SIRPH Analysis

The site-specific methylation measurements at selected CpG sites were done by the SIRPH method as previously described [22], [23]. The primers used for primer extension are given in Table S1. We selected one or two CpG sites for every region. The regions were selected in CpG rich sequences in the promoter region of the gene of interest (Figure S1). Detailed individual methylation values can be found in Table S2, while typical SIRPH chromatograms are shown in figure S2.

### Statistical Analysis

Descriptive analyses were performed for age and gender among cancer cases and controls and for clinical characteristics of the pancreatic cancer cases. Differences in methylation level values between cancer cases and controls were analyzed by Mann-Whitney and Wilcoxon test for related samples. The two-sided χ^2^ test was used to determine associations between the methylation statuses with various clinical-pathological features. Odds ratios (ORs) were obtained from the 2×2 contingency table comparing binary clinical-pathological variables and methylation level of site specific CpG. To evaluate the effects of site specific CpG methylation levels on case-control status, while controlling for intervariable confounding, logistic regression was performed to determine odds ratios and their associated 95% confidence intervals. In this analysis, site-specific CpG relative methylation level was broken into terciles based on the distribution in controls. Spearman’s correlation calculation was used to analyze correlation between all site-specific CpG methylation values. Survival analysis was performed in accordance with the Kaplan-Meier method, using the survival time starting from the day of surgery of the primary tumor to the date of death due to cancer (event) or to the last day of clinical follow-up (censored). Survival differences among comparator groups were analyzed by the log-rank test. *P*<0.05 was considered as significant. Data were analyzed using SPSS statistical software, version 17.0.0.

## Supporting Information

Figure S1 **Wild type and bisulfite converted sequences of studied regions with positioned PCR and SIRPH primers.**
(PDF)

Figure S2 **HPLC chromatograms samples of investigated CpGs.** First peak indicates primer, second peak, ddCTP and third peak, ddTTP extended oligos. Peak height is automatically calculated by WAVEMAKERTM software (Transgenomic).
(PDF)

Table S1 **Specific primers used for methylation-specific polymerase chain reaction (Bi) and SIRPH (SN) analysis.**
(PDF)

Table S2 **Raw data of methylation percentage of pancreatic ductal adenocarcinoma patients (blood: patient peripheral blood; tissue: patient tumor tissue) and control group individuals.**
(XLS)
