# Supplementary material for: Whole Blood DNA Aberrant Methylation in Pancreatic Adenocarcinoma Shows Association with the Course of the Disease: A Pilot Study
Source: PLoS One. 2012 May 22;7(5):e37509. doi: 10.1371/journal.pone.0037509 (PMC3358256; doi:10.1371/journal.pone.0037509)
Supplement: Figure S1 — Wild type and bisulfite converted sequences of studied regions with positioned PCR and SIRPH primers. (PDF) [file pone.0037509.s001.pdf]

Figure S1. Wild type and bisulfite converted sequences of studied regions with positioned PCR and SIRPH primers.

P16 (cyclin-dependent kinase inhibitor 2A) on NT_008413 (*Genbank accession number*)

Wild type sequence

21954841 ccactgcact ccagcctggg **CG**acagagaa agactc**CG**tc tcaaaaaaaa aaaaaaaaaa
21954901 aaaaaagttg ttttctgcta tttcctgaac tttatta**CG**t aaatgagaca **CG**tgagatct
21954961 ggaaggaggt ggaggtgaag accctcccac ctggcctgca tccagagtac ttggggtgtg
21955021 gcactgg**CG**t gggccccagg aaggatccca gccagttttg gtgctggagg cc**CG**gccaaa
21955081 ggaagggctg ctctcc**CG**tc c**CGCG**ggctc **CG**aggt**CG**c**C G**cacc**CG**gg**C GCG**cctgggc
21955141 ct**CG**tcacc**C GCG**ct**CG**tga **CGCG**tgctta caaaggaaac ttttacaggt tt**CG**gtgggc
21955201 agggcctttt attgcccttc tgctccacag cccc**CG**tgct taat**CG**gttg gttgggaggt
21955261 ttcttt**CG**tc **CG**tggttttg gaaagtccc**C G**cctccaaac **CG**cagtcc**CG** cagtcagttc
21955321 tgcagcctca gggcct**CG**aa cagccatgct tagaatc**CG**t ccttctttcc tgctccatct
21955381 acccttcccc tctggcttct tttttttcac tggagaaagc ccacc**CG**agt ctctcagcct
21955441 gctt**CG**gagg ttactg**CG**gc ctcctcca**CG** ggtagttctg ggacc**CG**ggt ctagtc**CGCG**

Bisulfite converted sequence

21954841 ttattgtatt ttagtttggg **CG**atagagaa agattt**CG**tt ttaaaaaaaa aaaaaaaaaa
21954901 aaaaaagttg ttttttgtta ttttttgaat tttatta**CG**t aaatgagata **CG**tgagattt
21954961 ggaaggaggt ggaggtgaag atttttttat ttggtttgta tttagagtat ttggggtgtg
Bi-p16-F1
21955021 gtattgg**CG**t gggttttagg aaggatttta gttagttttg gtgttggagg tt**CG**gttaaa
21955081 ggaagggttg tttttt**CG**tt t**CGCG**ggttt **CG**aggt**CG**t**C G**tatt**CG**gg**C GCG**tttgggt
SN-2
21955141 tt**CG**ttatt**C GCG**tt**CG**tga **CGCG**tgttta taaaggaaat ttttataggt tt**CG**gtgggt
Bi-p16-R1
21955201 agggtttttt attgtttttt tgttttatag tttt**CG**tgtt taat**CG**gttg gttgggaggt
21955261 tttttt**CG**tt **CG**tggttttg gaaagtttt**C G**tttttaaat **CG**tagttt**CG** tagttagttt
21955321 tgtagtttta gggttt**CG**aa tagttatgtt tagaatt**CG**t tttttttttt tgttttattt
21955381 attttttttt tttggttttt ttttttttat tggagaaagt ttatt**CG**agt tttttagttt
21955441 gttt**CG**gagg ttattg**CG**gt ttttttta**CG** ggtagttttg ggatt**CG**ggt ttagtt**CGCG**

SIRPH Primer P16 SN-2 (-2585 bp from the transcription start site)

APC (adenomatosis polyposis coli) on NT_034772 (*Genbank accession number*)

Wild type sequence

14488201 ttctctgtgc tgcaaaaatc atagcaat**CG** agatgtaatt tattactctc cctcccacct
14488261 c**CG**gcatctt gtgctaatcc ttctgccctg **CG**gacctccc c**CG**actcttt actatg**CG**tg
14488321 tcaactgcca tcaacttcct tgcttgctgg ggactggggc **CGCG**agggca tacccc**CG**ag
14488381 gggta**CG**ggg ctagggctag gcaggctgtg **CG**gttggg**CG** gggccctgtg ccccactg**CG**
14488441 gagtg**CG**ggt **CG**ggaag**CG**g agagagaagc agctgtgtaa tc**CG**ctggat g**CG**gaccagg
14488501 g**CG**ctcccca ttcc**CG**t**CG**g gagcc**CG**c**CG** attggctggg tgtggg**CG**ca **CG**tgac**CG**ac
14488561 atgtggctgt attggtgcag cc**CG**ccaggg tgtcactgga gacagaatgg aggtgctgc**C**
14488621 **G**gact**CG**gaa atggggtagg tgctggagcc accatggcca ggcttgctg**C G**gggggaggg
14488681 gggaaggtgg ttttccct**CG** cactgtctta aac**CG**atggc ctttccttgg cacagggtcc
14488741 actgcagcat gccaaa**CG**ag gaggcagggg **CG**t**CG**tcccc c**CG**cccccca ctgcagcact
14488801 ggagatggat ttcctgtact t**CG**gatccag ggtttttgac agaagaggaa gaagggggag

Bisulfite converted sequence

14488201 ttttttgtgt tgtaaaaatt atagtaat**CG** agatgtaatt tattattttt ttttttattt
14488261 t**CG**gtatttt gtgttaattt ttttgttttg **CG**gatttttt t**CG**atttttt attatg**CG**tg
14488321 ttaattgtta ttaatttttt tgtttgttgg ggattggggt **CGCG**agggta tatttt**CG**ag

Bi-APC-F1

14488381 gggta**CG**ggg ttagggttag gtaggttgtg **CG**gttggg**CG** gggttttgtg ttttattg**CG**

SN-1

14488441 gagtg**CG**ggt **CG**ggaag**CG**g agagagaagt agttgtgtaa tt**CG**ttggat g**CG**gattagg

SN-2

14488501 g**CG**tttttta tttt**CG**t**CG**g gagtt**CG**t**CG** attggttggg tgtggg**CG**ta **CG**tgat**CG**at

Bi-APC-R1

14488561 atgtggttgt attggtgtag tt**CG**ttaggg tgttattgga gatagaatgg aggtgttgt**C**
14488621 **G**gatt**CG**gaa atggggtagg tgttggagtt attatggtta ggtttgttg**C G**gggggaggg
14488681 gggaaggtgg tttttttt**CG** tattgtttta aat**CG**atggt ttttttttgg tatagggttt
14488741 attgtagtat gttaaa**CG**ag gaggtagggg **CG**t**CG**ttttt t**CG**tttttta ttgtagtatt
14488801 ggagatggat tttttgtatt t**CG**atttag ggtttttgat agaagaggaa gaagggggag

SIRPH Primer SN-1 (-131 bp from the transcription start site) and SN-2 (-75 bp from the transcription start site)

3OST2 (HS3ST2, Hepara sulfat-D-glucosaminyl-3-0-sulfotransferase-2) on NT_010393

(*Genbank accession number*)

Wild type sequence

```
14138701 GCGctcCGaa ccCGgCGcaC Gtaagagcct gggagCGccC GagcCGccCG gctgccCGga
14138761 gccccatCGc ctaggacCGg gagatgctgg aaatgcaacC Gcctgttccc CGaggagcCG
14138821 ctgccccCGg gaccccctgg cactgtgCGc accctggtca gcagccccCG gagaagaCGg
14138881 CGcccccaaC GccCGaccCG CGtggcCGtg gcagCGccaC GCGagccctc taggCGacCG
14138941 cagggccaca gcagctcagc CGcCGgtgcc ccctCGgaaa ccatgacccc CGgCGCGggc
14139001 ccatggagcc atggcctata gggtcctggg cCGCGCGggg ccacctcagc CGCGgagggC
14139061 GCGcaggctg ctcttCGcct tcaCGctctC Gctctcctgc acttacctgt gttacagctt
14139121 cctgtgctgc tgCGaCGacc tgggtCGgag cCGcctcctC GgCGCGcctC GctgcctcCG
14139181 CGgccccagC GCGggCGgcc agaaacttct ccagaagtcc CGcccctgtg atccctcCGg
14139241 gcCGaCGccc agCGagccca gCGctcccag CGCGccCGcC GcCGcCGtgc cCGcccctCG
14139301 cctctcCGgt tccaaccact cCGgctcacc caagctgggt accaagCGgt tgccccaagc
14139361 cctcattgtg ggCGtgaaga agggggcac cCGggcCGtg ctggagttta tcCGagtaca
14139421 ccCGgaCGtg CGggccttgg gcaCGgaacc ccacttcttt gacaggaact aCGgcCGCGg
14139481 gctggattgg tacaggtaag gaccaggagc tcCGctcCGt gCGcCGggtc tctgatCGct
```

Bisulfite converted sequence

```
14138701 GCGtttCGaa ttCGgCGtaC Gtaagagttt gggagCGttC GagtCGttCG gttgttCGga
14138761 gttttatCGt ttaggatCGg gagatgttgg aaatgtaatC Gtttgttttt CGaggagtCG
14138821 ttgttttCGg gatttttgg tattgtgCGt attttggtta gtagttttCG gagaagaCGg
14138881 CGtttttaaC GttCGattCG CGtggtCGtg gtagCGttaC GCGagttttt taggCGatCG
14138941 tagggttata gtagtttagt CGtCGgtgtt ttttCGgaaa ttatgatttt CGgCGCGggt
                   Bi-3OST2-F2                                SN-1
14139001 ttatggagtt atggtttata gggttttggg tCGCGCGggg ttattttagt CGCGgagggC
                 SN-2
14139061 GCGtaggttg tttttCGttt ttaCGttttC Gtttttttgt atttatttgt gttatagttt

14139121 tttgtgttgt tgCGaCGatt tgggtCGgag tCGtttttC GgCGCGtttC GttgttttCG
                                 Bi-3OST2-R2
14139181 CGgttttagC GCGggCGgtt agaaattttt ttagaagttt CGtttttgtg atttttCGg
14139241 gtCGaCGttt agCGagttta gCGtttttag CGCGttCGtC GtCGtCGtgt tCGtttttCG
14139301 ttttttCGgt tttaattatt tCGgtttatt taagttgggt attaagCGgt tgttttaagt
14139361 ttttattgtg ggCGtgaaga agggggtat tCGggtCGtg ttggagttta ttCGagtata
14139421 ttCGgaCGtg CGggttttgg gtaCGgaatt ttattttttt gataggaatt aCGgtCGCGg
14139481 gttggattgg tataggtaag gattaggagt ttCGtttCGt gCGtCGggtt tttgatCGtt
14139541 tttattggga gagttattCG tttttgtgt ttttttttt ttttaattta atttattgta
```

SIRPH Primer SN-1 (+99 bp from the transcription start site) and SN-2 (+124 bp from the transcription start site)

DAP-KINASE (DEATH ASSOSIATED PROTEIN KINASE 1)

on NT_023935 (*Genbank accession number*)

Wild type sequence

```
19278181 tggggaCGcC GacctCGggg tgcCGtggtg ccCGgcccca CGCGCGCGCG gggctgaggg
19278241 gtCGggggCG tccctggcCG cccagcttta acaaagggtg ctcctctcca cccCGCGagg
19278301 aggggcagct cCGgagaccC GgtcttcagC GagCGgggtc ttagCGcCGg ggaggtctac
19278361 ttccttttgg ggttgccatt ttactattat tattgccttt tttttttctt caaaaggact
19278421 ggagactgat gcatgagggg gctaCGgagg CGcaggagCG gtggtgatgg tctgggaagC
19278481 Ggagctgaag tgccctgggc tttggtgagg CGtgacagtt tatcatgacC Gtgttcaggc
19278541 aggaaaaCGt ggatgattac taCGacacCG gCGaggaact tggcaggtaa aggggtacc
19278601 agaagCGtac cctcctggat tgtggaaatg cataaCGatg gggccattgg gtggtaaaca
19278661 aatgcagttt gaatcaggCG tctccctCGc cctttctgga gatgCGcaaa tcatagagaa
19278721 aagagttact aacccagCGg taaacCGcct gatccaaggg cctgggggtg gaggagaggc
```

Bisulfite converted sequence

```
19278181 tggggaCGtC GatttCGggg tgtCGtggtg ttCGgtttta CGCGCGCGCG gggttgaggg
19278241 gtCGggggCG tttttggtCG tttagtttta ataaagggtg ttttttttta tttCGCGagg
19278301 aggggtagtt tCGgagattC GgtttttagC GagCGgggtt ttagCGtCGg ggaggtttat
                                                          Bi-DAPK-1-F1
19278361 ttttttttgg ggttgttatt ttattattat tattgttttt tttttttttt taaaaggatt

19278421 ggagattgat gtatgagggg gttaCGgagg CGtaggagCG gtggtgatgg tttgggaagC

19278481 Ggagttgaag tgttttgggt tttggtgagg CGtgatagtt tattatgatC Gtgtttaggt
                          SN-3                                Bi-DAPK-1-R1
19278541 aggaaaaCGt ggatgattat taCGatatCG gCGaggaatt tggtaggtaa aggggtatt
19278601 agaagCGtat ttttttggat tgtggaaatg tataaCGatg gggttattgg gtggtaaata
19278661 aatgtagttt gaattaggCG tttttttCGt tttttttgga gatgCGtaaa ttatagagaa
19278721 aagagttatt aatttagCGg taaatCGttt gatttaaggg tttgggggtg gaggagaggt
```

SIRPH Primer SN-3 (+1262 bp from the transcription start site)

ACIN1 (apoptotic chromatin condensation inducer 1) on NT_026437.11 (*Genbank accession number*)

Wild type sequence

```
4526761 ctgggctgCG gggCGcatac agatgCGgCG cccctccCGg acCGggccCG cagCGccCGg
4526821 cagggcactC GggCGggagg aCGtccaggc caCGgCGcca ggccatttcC Gaagagctgg
4526881 CGgggCGggt gcaccCGgga gCGCGaagct gccagtcccC GccCGcagCG gaaaaCGggC
4526941 gagCGCGCGg tgaggtgggg aattCGgagc ccactgCGga gagaccCGgc tcCGggctcc
4527001 CGgttccCGg ctggcttttg ggactggtgt ggatggaatc ccagctgcaa ggCGgCGgCG
4527061 ttttgagagc ccatgcaaag caCGgtagca ggtcagctgg gggacactta aggaCGgcag
4527121 aagggtccag agagtcCGag gccCGggccc agggagCGgg tCGggaacag aggcaCGaca
4527181 tCGgCGggag gtCGgaggCG ggcagtggaa cccttcttgg gaagctCGgt tacaggggcc
4527241 aacagagCGa ggagctgcCG ccccctggtg gtagttgtgg gaggcaccaa ggtggcaggc
4527301 aggtgaccaa acccctaatt taacCGtgca caggaccagg aaccagaaga ggacccaccc
4527361 tcctagccct gctggggtgc CGccaCGgac acaggttgct ccatgccttc cCGaatgagg
4527421 aaggtccaca gctttgggtt ggtcagggcc aaggcagggg ataaaagaCG tgggaagttc
4527481 aataagcagg aagcagtcag cctaaaggaa cacatggaat gagagcaact cctactccag
```

Bisulfite converted sequence

```
4526761 ttgggttgCG gggCGtatat agatgCGgCG tttttttCGg atCGggttCG tagCGttCGg
4526821 tagggtattC GggCGggagg aCGtttaggt taCGgCGtta ggttattttC Gaagagttgg
4526881 CGgggCGggt gtattCGgga gCGCGaagtt gttagttttC GttCGtagCG gaaaaCGggC
4526941 gagCGCGCGg tgaggtgggg aattCGgagt ttattgCGga gagattCGgt ttCGggtttt
                                        Bi-ACIN1-F2
4527001 CGgttttCGg ttggtttttg ggattggtgt ggatggaatt ttagttgtaa ggCGgCGgCG
                   SN-1
4527061 ttttgagagt ttatgtaaag taCGgtagta ggttagttgg gggatattta aggaCGgtag

4527121 aagggtttag agagttCGag gttCGggttt agggagCGgg tCGggaatag aggtaCGata
                                         SN-3
4527181 tCGgCGggag gtCGgaggCG ggtagtggaa tttttttttgg gaagttCGgt tataggggtt
                                                          Bi-ACIN1-R2
4527241 aatagagCGa ggagttgtCG tttttggtg gtagttgtgg gaggtattaa ggtggtaggt
4527301 aggtgattaa atttttaatt taatCGtgta taggattagg aattagaaga ggatttattt
4527361 ttttagtttt gttggggtgt CGttaCGgat ataggttgtt ttatgttttt tCGaatgagg
4527421 aaggtttata gttttgggtt ggttagggtt aaggtagggg ataaaagaCG tgggaagttt
4527481 aataagtagg aagtagttag tttaaaggaa tatatggaat gagagtaatt tttattttag
```

SIRPH Primer SN-1(-548 bp from the transcription start site) and SN-3(-415 bp from the transcription start site)

BCL2 (antagonist of cell death) on NT_033903 (*Genbank accession number*)

Wild type sequence

```
9342301 tcccaccttc ctgactctag ctggtgtcca ctgagggaca cactccaagt cagttcccag
9342361 gtcctcaaat accagctgaa tgtctaactt ctgggtgtga cattccagcc cctagaagcc
9342421 tggcctctgc tgctcccctg tccttccatg ggtccactcc tccttgCGcC Gggggggatg
9342481 accctcacct caccttcCGg tCGcagctct agctgcctcc tccctgaagc cctctctgac
9342541 tgccctgcCG attcCGggcc ctCGggagct tcCGCGagca gggcCGtggC Gcaatgcatc
9342601 tctgtgccCG gcaccaagaa cCGgccCGgg ttatagggggg CGccaataaa tctttctgga
9342661 aCGgagcccc CGctctgtcC GggtcccCGc atgaagcctc agtttccagC GacCGCGctg
9342721 ctgccagcct ggctcagcCG ctccctccCG cccCGCGagg taccCGggCG gCGctggccc
9342781 CGggcCGgcc acacagtagc tgctggaaag ggctgaccca aataggagCG agtgaccctc
9342841 tctgggggc CGCGgcaCGg tttgcagagC GCGggatCGg gctgcCGgca gCGctgCGgt
9342901 cccaatgCGC Gtgctgggga cttggacctg ggcCGCGcag cccaggccCG CGagcCGcCG
9342961 CGaccccttc CGtctctgtC Gctggatgtc aggcagagcc taagtttccc tCGtaagaca
```

Bisulfite converted sequence

```
9342301 ttttattttt ttgattttag ttggtgttta ttgagggata tattttaagt tagtttttag

9342361 gtttttaaat attagttgaa tgtttaattt ttgggtgtga tattttagtt tttagaagtt
9342421 tggtttttgt tgtttttttg ttttttatg ggtttatttt tttttgCGtC Gggggggatg
                                                        Bi-BCL2-F1
9342481 atttttattt tatttttCGg tCGtagtttt agttgttttt tttttgaagt tttttttgat
                                                              SN-1
9342541 tgttttgtCG atttCGggtt ttCGggagtt ttCGCGagta gggtCGtggC Gtaatgtatt

9342601 tttgtgttCG gtattaagaa tCGgttCGgg ttataggggg CGttaataaa tttttttgga

9342661 aCGgagtttt CGttttgttC GggttttCGt atgaagtttt agtttttagC GatCGCGttg
9342721 ttgttagttt ggtttagtCG ttttttttCG tttCGCGagg tattCGggCG gCGttggttt
                      Bi-BCL2-R1
9342781 CGggtCGgtt atatagtagt tgttggaaag ggttgattta aataggagCG agtgattttt
9342841 tttgggggt CGCGgtaCGg tttgtagagC GCGggatCGg gttgtCGgta gCGttgCGgt
9342901 tttaatgCGC Gtgttgggga tttggatttg ggtCGCGtag tttaggttCG CGagtCGtCG
9342961 CGattttttt CGttttgtC Gttggatgtt aggtagagtt taagtttttt tCGtaagata
```

SIRPH Primer SN-1 (-496 bp from the transcription start site).

CD44 on NT_009237 (*Genbank accession number*)

Wild type sequence

33947641 **CG**c**CGCG**ggg ccag**CG**ggag aagaaagcca gtg**CG**tctct ggg**CG**caggg gccagtgggg
33947701 ct**CG**gaggca caggcaccc**C GCG**acactcc aggttccc**CG** accca**CG**tcc ctggcagccc
33947761 **CG**attattta cagcctcagc agagca**CG**gg g**CG**ggggcag aggggcc**CG**c c**CG**ggagggc
33947821 tgctacttct taaaacctct g**CG**ggctgct tagtcacagc cccccttgct tgggtgtgtc
33947881 ctt**CG**ct**CG**c tccctccctc **CG**tcttaggt cactgttttc aacct**CG**aat aaaaactgca
33947941 gccaacttc**C G**aggcagcct cattgcccag **CG**gaccccag cctctgccag gtt**CG**gtc**CG**
33948001 ccatcct**CG**t cc**CG**tcctc**C G**c**CG**gcccct gccc**CGCG**cc cagggatcct ccagctcctt
33948061 t**CG**cc**CGCG**c cctc**CG**tt**CG** ctc**CG**gacac catggacaag ttttggtggc a**CG**cagcctg
33948121 gggactctgc ct**CG**tgc**CG**c tgagcctgg**C G**cagat**CG**gt gagtgcc**CG**c **CG**cagcctgg
33948181 gcagcaagat gggtg**CG**ggg tgctcag**CGC G**gacc**CG**g**CG** gcagcccctc **CG**gctgagtc
33948241 ggccctgggg gactggagtc aagtgagctg tctg**CG**aagt gcattgggct c**CG**gaaagca
33948301 gggctgggat ttg**CG**ctaaa c**CG**ttggaga atgtgtctgt ggaagcacca tttggttgaa

Bisulfite converted sequence

33947641 **CG**t**CGCG**ggg ttag**CG**ggag aagaaagtta gtg**CG**ttttt ggg**CG**taggg gttagtgggg
33947701 tt**CG**gaggta taggtattt**C GCG**atatttt aggtttt**CG** attta**CG**ttt ttggtagttt
33947761 **CG**attattta tagttttagt agagta**CG**gg g**CG**ggggtag aggggtt**CG**t t**CG**ggagggt

Bi-CD44-F1

33947821 tgttattttt taaaattttt g**CG**ggttgtt tagttatagt tttttttgtt tgggtgtgtt
33947881 ttt**CG**tt**CG**t tttttttttt **CG**ttttaggt tattgttttt aattt**CG**aat aaaaattgta

SN-2

33947941 gttaatttt**C G**aggtagttt tattgtttag **CG**gattttag tttttgttag gtt**CG**gtt**CG**
33948001 ttatttt**CG**t tt**CG**ttttt**C G**t**CG**gttttt gttt**CGCG**tt tagggatttt ttagtttttt

Bi- CD44-R1

33948061 t**CG**tt**CGCG**t tttt**CG**tt**CG** ttt**CG**gatat tatggataag ttttggtggt a**CG**tagtttg
33948121 gggattttgt tt**CG**tgt**CG**t tgagtttgg**C G**tagat**CG**gt gagtgtt**CG**t **CG**tagtttgg
33948181 gtagtaagat gggtg**CG**ggg tgtttag**CGC G**gatt**CG**g**CG** gtagtttttt **CG**gttgagtt
33948241 ggttttgggg gattggagtt aagtgagttg tttg**CG**aagt gtattgggtt t**CG**gaaagta
33948301 gggttgggat ttg**CG**ttaaa t**CG**ttggaga atgtgtttgt ggaagtatta tttggttgaa

SIRPH Primer SN-2 (+320 bp from the transcription start site)

RARB (RETINOIC ACID RECEPTOR, β) on NT_022517 (*Genbank accession number*)

Wild type sequence

25409521 agcacttctt gtattgtttt taaggtgaga aataggaaag aaaa**CG**c**CG**g cttgtg**CG**ct
25409581 **CG**ctgcctgc ctctctggct gtctgctttt gcagggctgc tgggagtttt taagctctgt
25409641 gagaatcctg ggagttggtg atgtcagact agttgggtca tttgaaggtt agcagcc**CG**g
25409701 gtagggttca c**CG**aaagttc act**CG**catat attaggcaat tcaatctttc attctgtgtg
25409761 acagaagtag taggaagtga gctgttcaga ggcaggaggg tctattcttt gccaaagggg
25409821 ggaccagaat tcccccatg**C G**agctgtttg aggactggga tgc**CG**agaa**C GCG**ag**CG**atc
25409881 **CG**agcagggt ttgtctgggc ac**CG**t**CG**ggg taggatc**CG**g aa**CG**catt**CG** gaaggctttt
25409941 tgcaagcatt tacttggaag gagaacttgg gatctttctg ggaacccccC **G**ccc**CG**gctg
25410001 gattggc**CG**a gcaagcctgg aaaatggtaa atgatcattt ggatcaatta caggctttta
25410061 gctggcttgt ctgtcataat tcatgatt**CG** gggctgggaa aaagaccaac agccta**CG**tg
25410121 ccaaaaaagg ggcagagttt gatggagttg ggtggacttt tctatgccat ttgcctccac
25410181 acctagagga taagcacttt tgcagacatt cagtgcaagg gagatcatgt ttgactgtat

Bisulfite converted sequence

25409521 agtatttttt gtattgtttt taaggtgaga aataggaaag aaaa**CG**t**CG**g tttgtg**CG**tt
25409581 **CG**ttgtttgt tttttggtt gtttgttttt gtagggttgt tgggagtttt taagttttgt
25409641 gagaatttg ggagttggtg atgttagatt agttgggtta tttgaaggtt agtagtt**CG**g
25409701 gtagggttta t**CG**aaagttt att**CG**tatat attaggtaat ttaatttttt attttgtgtg

Bi-RARBeta-F1

25409761 atagaagtag taggaagtga gttgtttaga ggtaggaggg tttatttttt gttaaagggg

SN-1

25409821 ggattagaat tttttatg**C G**agttgtttg aggattggga tgt**CG**agaa**C GCG**ag**CG**att

SN-2

25409881 **CG**agtagggt ttgtttgggt at**CG**t**CG**ggg taggatt**CG**g aa**CG**tatt**CG** gaaggttttt
25409941 tgtaagtatt tatttggaag gagaatttgg gatttttttg ggaattttt**C G**ttt**CG**gttg

Bi-RARBeta-R1

25410001 gattggt**CG**a gtaagtttgg aaaatggtaa atgattattt ggattaatta taggttttta
25410061 gttggtttgt ttgttataat ttatgatt**CG** gggttgggaa aaagattaat agttta**CG**tg
25410121 ttaaaaaagg ggtagagttt gatggagttg ggtggatttt tttatgttat ttgtttttat
25410181 atttagagga taagtatttt tgtagatatt tagtgtaagg gagattatgt ttgattgtat

SIRPH Primer SN-1 (+90 bp from the transcription start site) and SN-2(+125 bp from the transcription start site)

TNFRSF10C (tumor necrosis factor receptor superfamily, member 10c, decoy without an intracellular domain) on NT_023666 (*Genbank accession number*)

Wild type sequence

```
1334521 agcctggaca ggaccCGgaa tcaaacCGca ggccctgggt cacCGctgcC Ggaaagagcc
1334581 agttcctgtc CGtccatgca cccaccacca aaacccaggc cttcctggag gtgctagggg
1334641 aggccatgcc cctttctga gtgcttggaa gtgactgctg caagtgacaa gtgaccaCGc
1334701 cttttccccC GCGggtataa attcagaggC GctgCGctcC Gattctggca gtgcagctgt
1334761 gggaacctct ccaCGCGcaC Gaactcagcc aaCGatttct gatagatttt tgggagtttg
1334821 accagagatg caaggggtga aggagCGctt cctacCGtta gggaactctg gggacagagC
1334881 GcccCGgcCG cctgatggcC Gaggcagggt gCGacccagg acccaggaCG gCGtCGggaa
1334941 ccataccatg gccCGgatcc ccaagaccct aaagttCGtC GtCGtcatCG tCGCGgtcct
1335001 gctgccagtg agtccCGgcC GCGgtccctg gctggggaag agCGcacctg gCGcCGggag
1335061 ggggcaggga gaCGgggaca CGgcagggat gcctggccct ggtcacctgC GgcCGggcat
1335121 gtcCGggcag gaCGaactCG cCGtCGgagt caggggaaga actgggtccc CGggctgggc
1335181 aggagggacc CGgcCGCGag ggagcagaga ggCGgtcccc ctggctgccc CGagccCGCG
1335241 aagggaggga agttccagaa tCGagagagg gagggagtca aggtggaacc catagagtga
1335301 gcctcctgaa gacacagagC Ggttgcctct ctcattaatt aattaattag ttaataaaat
1335361 taaccccatg tttacattct taaaCGtgtt ccttggagat CGgtttaacc aacagccagt
1335421 gaaaaaactt ttcagCGctg tctttagcaa ctttcacctc ctctgtcctg aaagtggcag
```

Bisulfite converted sequence

```
1334881 GtttCGgtCG tttgatggtC Gaggtagggt gCGatttagg atttaggaCG gCGtCGggaa
1334941 ttatattatg gttCGgattt ttaagatttt aaagttCGtC GtCGttatCG tCGCGgtttt
1335001 gttgttagtg agtttCGgtC GCGgtttttg gttggggaag agCGtatttg gCGtCGggag
                                             Bi-TNFRSF10C-F1
1335061 ggggtaggga gaCGgggata CGgtagggat gtttggtttt ggttatttgC GgtCGggtat
                                                  Sn-1
1335121 gttCGggtag gaCGaattCG tCGtCGgagt taggggaaga attgggtttt CGggttgggt
1335181 aggagggatt CGgtCGCGag ggagtagaga ggCGgttttt ttggttgttt CGagttCGCG
                                                 Bi-TNFRSF10C-R1
1335241 aagggaggga agttttagaa tCGagagagg gagggagtta aggtggaatt tatagagtga

1335301 gtttttgaa gatatagagC Ggttgttttt tttattaatt aattaattag ttaataaaat
1335361 taattttatg tttatatttt taaaCGtgtt ttttggagat CGgtttaatt aatagttagt
1335421 gaaaaaattt tttagCGttg tttttagtaa tttttatttt ttttgttttg aaagtggtag
```

SIRPH Primer SN-1 (+400bp from the transcription start site).

Alu (accession numbers: Alu-J U14567; Alu-Sb U14568; Alu-Sb1 U14569; Alu-Sb2 U14570; Alu-Sc U14571; Alu-Sp U14572; Alu-Sq U14573; Alu-Sx U14574)

Wild type sequences

```
ps-alu      ----------ggcCGggCGCGgtggctcaCGcctgtaatcccagcactttgggaggcCGa 50
sq-alu      ----------ggcCGggCGCGgtggctcaCGcctgtaatcccagcactttgggaggcCGa 50
sx-alu      ggCGggCGaggcCGggCGCGgtggctcaCGcctgtaatcccagcactttggga------ 54
j-alu        ----------ggcCGggCGCGgtggctcaCGcctgtaatcccagcactttgggaggcCGa 50
sb1-alu      ----------ggcCGggCGCGgtggctcaCGcctgtaatcccagcactttgggaggcCGa 50
sb2-alu      ----------ggcCGggCGCGgtggctcaCGcctgtaatcccagcactttgggaggcCGa 50
sb-alu       ----------ggcCGggCGCGgtggctcaCGcctgtaatcccagcactttgggaggcCGa 50
sc-alu       ----------ggcCGggCGCGgtggctcaCGcctgtaatcccagcactttgggaggcCGa 50

sp-alu      ggCGggCGgatcacctgaggtCGggagttCGagaccagcctgaccaacatggagaaaccc 110
sq-alu      ggCGggtggatcacctgaggtcaggagttCGagaccagcctggccaacatggtgaaaccc 110
sx-alu      ----ggaagatcacctgaggtcaggagttCGagaccagcctggccaacatggtgaaaccc 110
j-alu       ggCGggaggatcacttgagcccaggagttCGagaccagcctgggcaacatagtgaaaccc 110
sb1-alu     ggCGggCGgatca--CGaggtcaggagatCGagaccatccCGgctaaaaCGgtgaaaccc 108
sb2-alu     ggCGggtggatca--tgaggtcaggagatCGagaccatcctggctaacaaggtgaaaccc 108
sb-alu      ggCGggCGgatca--CGaggtcaggagatCGagaccatcctggctaacaCGgtgaaaccc 108
sc-alu      ggCGggCGgatca--CGaggtcaagagatCGagaccatcctggccaacatggtgaaaccc 108

sp-alu      CGtctctactaaaaatacaaaaa-ttagcCGggCGtggtggCGcatgcctgtaatcccag 169
sq-alu      CGtctctactaaaaatacaaaaa-ttagcCGggCGtggtggCGggCGcctgtaatcccag 169
sx-alu      CGtctctactaaaaatacaaaaa-ttagcCGggCGtggtggCGCGCGcctgtaatcccag 169
j-alu       CGtctctacaaaaaatacaaaaa-ttagcCGggCGtggtggCGCGCGcctgtagtcccag 169
sb1-alu     CGtctctactaaaaatacaaaaa-ttagcCGggCGtagtggCGggCGcctgtagtcccag 167
sb2-alu     CGtctctactaaaaatacaaaaaattagcCGggCGCGgtggCGggCGcctgtagtcccag 168
sb-alu      CGtctctactaaaaatacaaaaa-ttagcCGggCGtggtggCGggCGcctgtagtcccag 167
sc-alu      CGtctctactaaaaatacaaaaa-ttagctgggCGtggtggCGCGCGcctgtagtcccag 167

sp-alu      ctactCGggaggctgaggcaggagaatCGcttgaaccCGggaggCGgaggttgCGgtgag 229
sq-alu      ctactCGggaggctgaggcaggagaatCGcttgaaccCGggaggCGgaggttgcagtgag 229
sx-alu      ctactCGggaggctgaggcaggagaatCGcttgaaccCGggaggCGgaggttgcagtgag 229
j-alu       ctactCGggaggctgaggcaggaggatCGcttgagccCGggaggtCGaggctgcagtgag 229
sb1-alu     ctacttgggaggctgaggcaggagaatggCGtgaaccCGggaggCGgagcttgcagtgag 227
sb2-alu     ctactggggaggctgaggcaggagaatggCGtgaaccCGggaagCGgagcttgcagtgag 228
sb-alu      ctactCGggaggctgaggcaggagaatggCGtgaaccCGggaggCGgagcttgcagtgag 227
sc-alu      ctactCGggaggctgaggcaggagaatCGcttgaaccCGggaggCGgaggttgcagtgag 227

sp-alu      cCGagatCGCGccattgcactcca-------gcctgggcaacaagagCGaaactcCGtct 282
sq-alu      cCGagatCGCGccactgcactcca-------gcctgggcaacaagagCGaaactcCGtct 282
sx-alu      cCGagatCGCGccactgcactcca-------gcctgggCGaca-gagCGagactcCGtct 281
j-alu       cCGtgatCGCGccactgcactcca-------gcctgggCGaca-gagCGagaccctgtct 281
sb1-alu     cCGagatccCGccactgcactcca-------gcctgggCGaca-gagCGagactcCGtct 279
sb2-alu     cCGagattgCGccactgcagtcCGcagtcCGgcctgggCGaca-gagCGagactcCGtct 287
sb-alu      cCGagatCGCGccactgcactcca-------gcctgggCGaca-gagCGagactcCGtct 279
sc-alu      cCGagatCGCGccactgcactcca-------gcctgg-CGaca-gagCGagactcCGtct 278


Sp-Alu      CAAAAAAAA 291
Sq-Alu      CAAAAAAAA 291
Sx-Alu      CAAAAAAAA 290
J-Alu       CAAAAAAAA 290
Sb1-Alu     CAAAAAAAA 288
Sb2-Alu     CAAAAAAAA 296
Sb-Alu      CAAAAAAAA 288
Sc-Alu      CAAAAAAAA 287
```

Bisulfite sequences

```
                                                      Bi-Alu-All-F3
Sp-Alu      ----------ggtCGggCGtggtggtttaCGtttgtaatttagtattttgggaggtCGa 50
sq-alu      ----------ggtCGggCGtggtggtttaCGtttgtaatttagtattttgggaggtCGa 50
sx-alu      ggCGggCGaggtCGggCGtggtggtttaCGtttgtaatttagtattttggga------ 54
j-alu       ----------ggtCGggCGtggtggtttaCGtttgtaatttagtattttgggaggtCGa 50
sb1-alu     ----------ggtCGggCGtggtggtttaCGtttgtaatttagtattttgggaggtCGa 50
sb2-alu     ----------ggtCGggCGtggtggtttaCGtttgtaatttagtattttgggaggtCGa 50
```

```
sb-alu      ----------ggtCGggCGtggtggtttaCGtttgtaatttagtatttgggaggtCGa 50
sc-alu      ----------ggtCGggCGtggtggtttaCGtttgtaatttagtatttgggaggtCGa 50


sp-alu      ggCGggCGgattatttgaggtCGggagttCGagattagtttgattaatatggagaaattt 110
sq-alu      ggCGggtggattatttgaggttaggagttCGagattagtttggttaatatggtgaaattt 110
sx-alu      ----ggaagattatttgaggttaggagttCGagattagtttggttaatatggtgaaattt 110
j-alu       ggCGggaggattatttgagtttaggagttCGagattagtttgggtaatatagtgaaattt 110
sb1-alu     ggCGggCGgatta--CGaggttaggagatCGagattatttCGgttaaaaCGgtgaaattt 108
sb2-alu     ggCGggtggatta--tgaggttaggagatCGagattattttggttaataaggtgaaattt 108
sb-alu      ggCGggCGgatta--CGaggttaggagatCGagattattttggttaataCGgtgaaattt 108
sc-alu      ggCGggCGgatta--CGaggttaagagatCGagattattttggttaatatggtgaaattt 108

                          SN-1                                  SN-3
sp-alu      CGtttttattaaaaatataaaaa-ttagtCGggCGtggtggCGtatgtttgtaattttag 169
sq-alu      CGtttttattaaaaatataaaaa-ttagtCGggCGtggtggCGggCGtttgtaattttag 169
sx-alu      CGtttttattaaaaatataaaaa-ttagtCGggCGtggtggCGCGCGtttgtaattttag 169
j-alu       CGtttttataaaaaatataaaaa-ttagtCGggCGtggtggCGCGCGtttgtagttttag 169
sb1-alu     CGtttttattaaaaatataaaaa-ttagtCGggCGtagtggCGggCGtttgtagttttag 167
sb2-alu     CGtttttattaaaaatataaaaaattagtCGggCGCGgtggtgggCGtttgtagttttag 168
sb-alu      CGtttttattaaaaatataaaaa-ttagtCGggCGtggtggCGggCGtttgtagttttag 167
sc-alu      CGtttttattaaaaatataaaaa-ttagttgggCGtggtggCGCGCGtttgtagttttag 167


sp-alu      ttattCGggaggttgaggtaggagaatCGtttgaattCGggaggCGgaggttgCGgtgag 229
sq-alu      ttattCGggaggttgaggtaggagaatCGtttgaattCGggaggCGgaggttgtagtgag 229
sx-alu      ttattCGggaggttgaggtaggagaatCGtttgaattCGggaggCGgaggttgtagtgag 229
j-alu       ttattCGggaggttgaggtaggaggatCGtttgagttCGggaggtCGaggttgtagtgag 229
sb1-alu     ttatttgggaggttgaggtaggagaatggCGtgaattCGggaggCGgagtttgtagtgag 227
sb2-alu     ttattggggaggttgaggtaggagaatggCGtgaattCGggaagCGgagtttgtagtgag 228
sb-alu      ttattCGggaggttgaggtaggagaatggCGtgaattCGggaggCGgagtttgtagtgag 227
sc-alu      ttattCGggaggttgaggtaggagaatCGtttgaattCGggaggCGgaggttgtagtgag 227
                          Bi-Alu-All-R2
sp-alu      tCGagatCGCGttattgtatttta-------gtttgggtaataagagtgaaatttCGttt 282
sq-alu      tCGagatCGCGttattgtatttta-------gtttgggtaataagagtgaaatttCGttt 282
sx-alu      tCGagatCGCGttattgtatttta-------gtttgggCGata-gagCGagatttCGttt 281
j-alu       tCGtgatCGCGttattgtatttta-------gtttgggCGata-gagCGagattttgttt 281
sb1-alu     tCGagatttCGttattgtatttta-------gtttgggCGata-gagCGagatttCGttt 279
sb2-alu     ttgagattgCGttattgtagttCGtagttCGgtttgggCGata-gagCGagatttCGttt 287
sb-alu      tCGagatCGCGttattgtatttta-------gtttgggCGata-gagCGagatttCGttt 279
sc-alu      tCGagatCGCGttattgtatttta-------gtttgg-CGata-gagCGagatttCGttt 278

Sp-Alu      TAAAAAAAA 291
Sq-Alu      TAAAAAAAA 291
Sx-Alu      TAAAAAAAA 290
J-Alu       TAAAAAAAA 290
Sb1-Alu     TAAAAAAAA 288
Sb2-Alu     TAAAAAAAA 296
Sb-Alu      TAAAAAAAA 288
Sc-Alu      TAAAAAAAA 287
```

SIRPH Primer SN-1, SN-3 and SN-4

LINE-1 elements (accession number X52235)

Wild type sequence

```
 61 CGcagaagac gggtgatttc tgcatttcca tctgaggtac tgggttcatc tcactaggga
121 gtgccagaca gtgggCGcag gtcagtgggt gCGCGcacCG tgcaCGagcC GaagcagggC
181 Gaggcattgt ctcacttggg aagCGcaagg ggtcagggag ttccctttcC Gagtcaaaga
241 aaggggtgaC GgaCGcacct ggaaaatCGg gtctctccca ccCGaatatt9 gCGctttCGg
301 acCGgcttaa aaaaCGgCGc acCGCGagat tatatcttgc acctggctag gagggtccta
361 CGcccaCGga gtctCGctga ttgctagcac agcagtctga gatcaaactg caaggcCGca
421 gcaaggctgg gggaggggCG ccCGccattg cccaggcttg cttgggtaaa caaagcagcc
481 tggcagctCG aactgggtgg agcccaccac agctcaagga ggcctgcctg cctttgtagg
```

Bisulfite sequence

```
                                                         Bi-LINE-1-F1
 61 CGtagaagat gggtgatttt tgtattttta tttgaggtat tgggtttatt ttattaggga
121 gtgttagata gtgggCGtag gttagtgggt gCGCGtatCG tgtaCGagtC GaagtagggC
181 Gaggtattgt tttatttggg aagCGtaagg ggttagggag tttttttttC Gagttaaaga
        SN-1                           SN-8
241 aaggggtgaC GgaCGtattt ggaaaatCGg gtttttttta ttCGaatatt gCGtttCGg

301 atCGgtttaa aaaaCGgCGt atCGCGagat tatatttgt atttggttag gagggtttta
361 CGtttaCGga gtttCGttga ttgttagtat agtagtttga gattaaattg taaggtCGta
421 gtaaggttgg gggaggggCG ttCGttattg tttaggtttg tttgggtaaa taaagtagtt
                         Bi-LINE-1-R1
481 tggtagttCG aattgggtgg agtttattat agtttaagga ggtttgtttg tttttgtagg
```

SIRPH Primer: SN-1 and SN-8.
