# Supplementary material for: Whole Blood DNA Aberrant Methylation in Pancreatic Adenocarcinoma Shows Association with the Course of the Disease: A Pilot Study
Source: PLoS One. 2012 May 22;7(5):e37509. doi: 10.1371/journal.pone.0037509 (PMC3358256; doi:10.1371/journal.pone.0037509)
Supplement: Figure S2 — HPLC chromatograms samples of investigated CpGs. First peak indicates primer, second peak, ddCTP and third peak, ddTTP extended oligos. Peak height is automatically calculated by WAVEMAKERTM software (Transgenomic). (PDF) [file pone.0037509.s002.pdf]

Supplemental figure S2. HPLC chromatograms samples of investigated CpGs. First peak indicates primer second- ddCTP and third- ddTTP extended oligos. Peak height is automatically calculated by the WAVEMAKER™ software (Transgenomic).

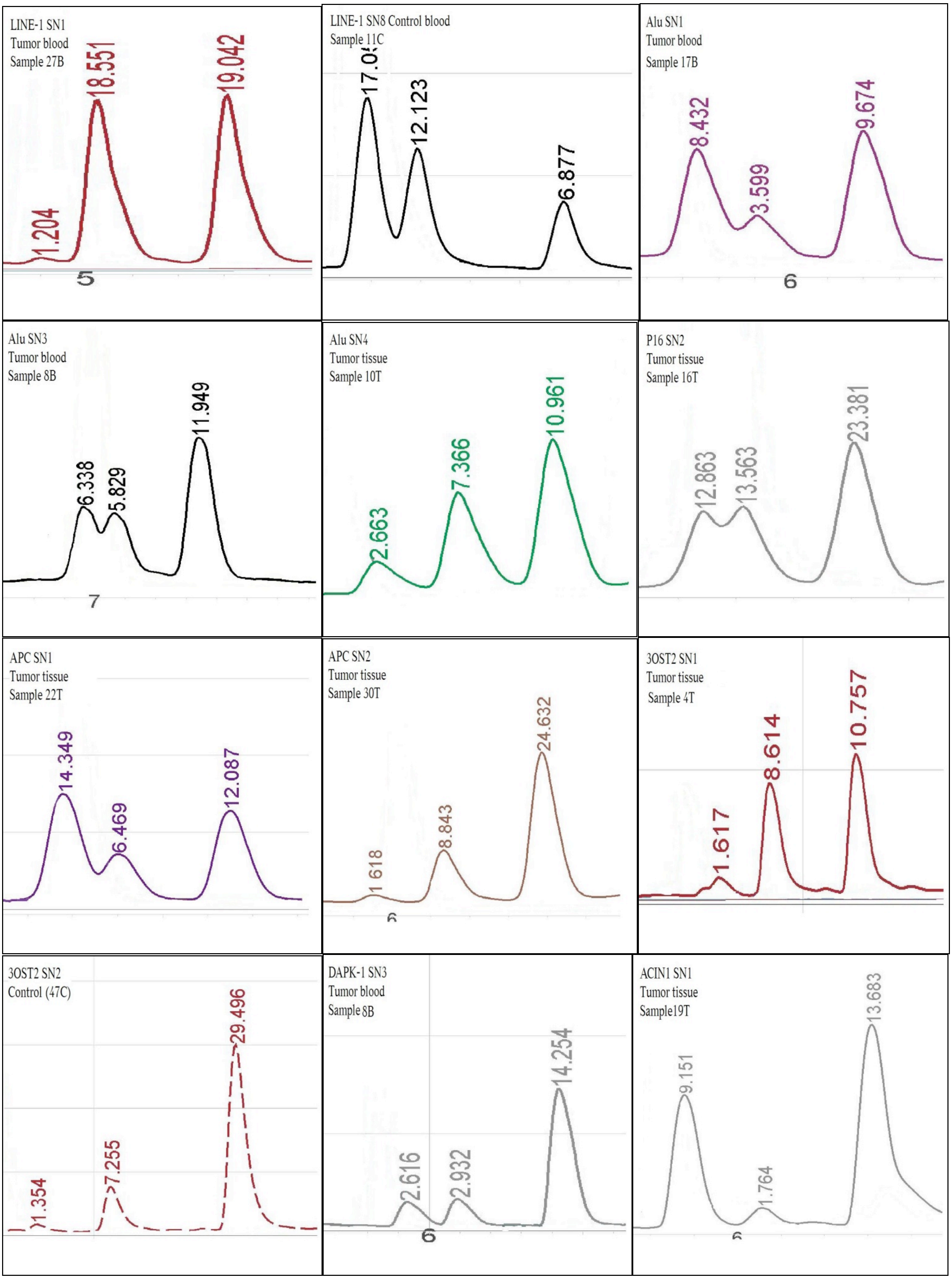

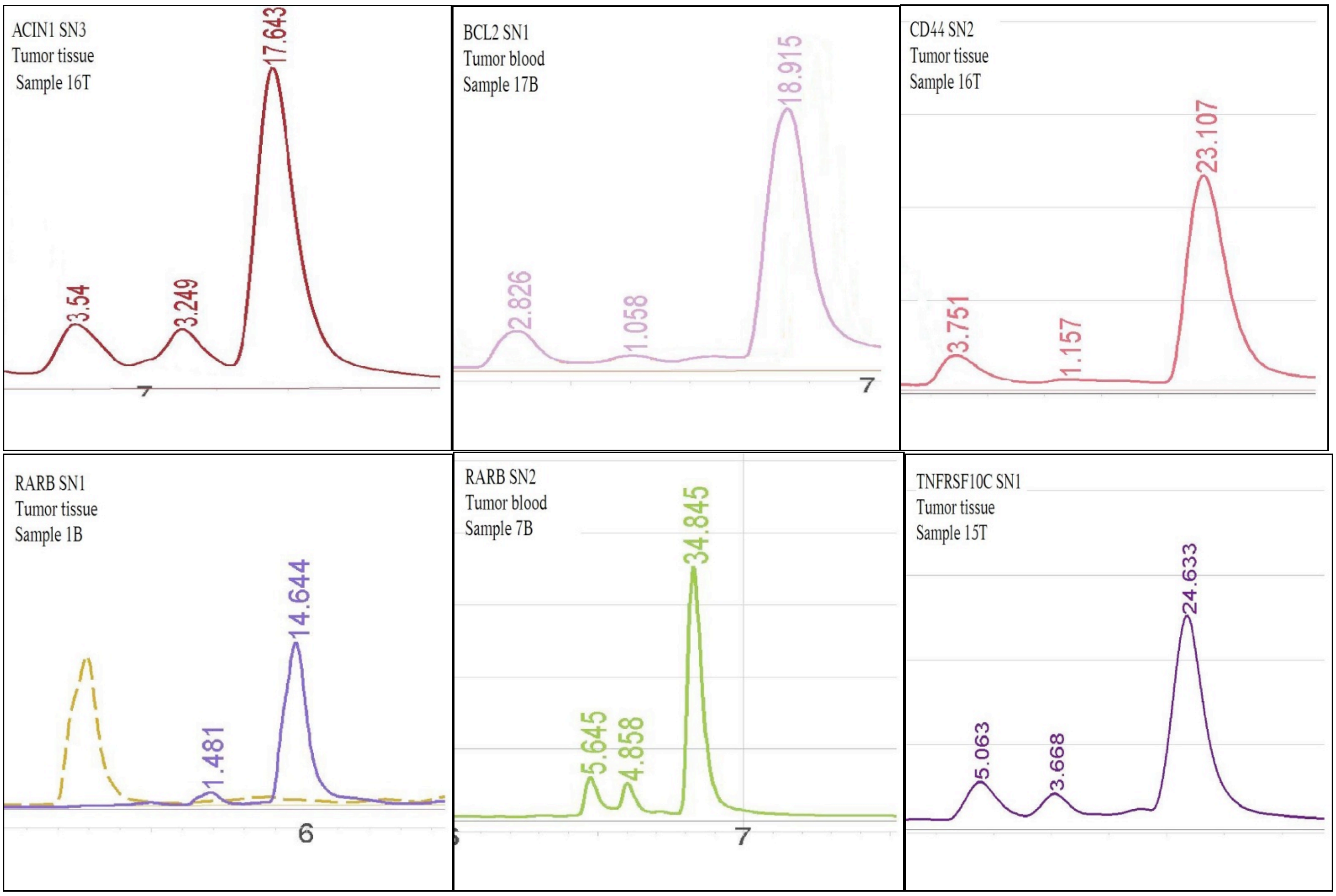

ACIN1 SN3
Tumor tissue
Sample 16T
3.54
3.249
17.643
7
BCL2 SN1
Tumor blood
Sample 17B
2.826
1.058
18.915
7
CD44 SN2
Tumor tissue
Sample 16T
3.751
1.157
23.107
RARB SN1
Tumor tissue
Sample 1B
1.481
14.644
6
RARB SN2
Tumor blood
Sample 7B
5.645
4.858
34.845
7
TNFRSF10C SN1
Tumor tissue
Sample 15T
5.063
3.668
24.633
