# Supplementary material for: Whole Blood DNA Aberrant Methylation in Pancreatic Adenocarcinoma Shows Association with the Course of the Disease: A Pilot Study
Source: PLoS One. 2012 May 22;7(5):e37509. doi: 10.1371/journal.pone.0037509 (PMC3358256; doi:10.1371/journal.pone.0037509)
Supplement: Table S1 — Specific primers used for methylation-specific polymerase chain reaction (Bi) and SIRPH (SN) analysis. (PDF) [file pone.0037509.s003.pdf]

Supplemental table S1. Specific primers used for methylation-specific polymerase chain reaction (Bi) and SIRPH (SN) analysis.

Region	Oligo name	Sequence	Genbank accession number	Bp	Annealing ^{0}C
Alu	Bi-Alu-All-F3	ttg taa ttt tag tat ttt gg	Consensus sequence	238	53
	Bi-Alu-All-R2	cca aac taa aat aca ata a			
	Alu SN-1	aat ata aaa att agt			
	Alu SN-3	ttt gta att tta gtt att			
	Alu SN-4	gag gtt gta gtg agt			
LINE-1	Bi-LINE-1-F1	att tta tta ggg agt gtt aga tag tgg g	Consensus sequence	410	59
	Bi-LINE-1-R1	tta aac tat aat aaa ctc cac cca at			
	SN-1	gaa agg ggt gat ga			
	SN-8	ggg ttt ttt tta tt			
P16	Bi-p16-F1	gat ttt agt tag ttt tgg tgt t	**NT_008413** 21955044	213	58
	Bi-p16-R1	aaa aaa acc tcc caa cca ac	21955266		
	SN-2	ttt ata aag gaa att ttt ata ggt tt			
APC	Bi-APC-F1	ggg tta ggg tta ggt agg ttg t	**NT_034772** 14488388	231	61
	Bi-APC-R1	aca cct cca ttc tat ctc caa taa c	14488618		
	SN-1	gaa gta gtt gtg taa tt			
	SN-2	att ggt tgg gtg tgg g			
3OST2	Bi-3OST2-F2	tgg agt tat ggt tta tag ggt ttt g	**NT_010393** 14139004	217	56
	Bi-3OST2-R2	aaa ctt cta aaa aaa ttt cta acc	14139220		
	SN-1	ggg tta ttt tag t			
	SN-2	gta ggt tgt ttt t			
ACIN1	Bi-ACIN1-F2	gga ttg gtg tgg atg gaa ttt tag ttg taa	**NT_026437.11** 4527021	286	61
	Bi-ACIN1-R2	tca cct acc tac cac ctt aat acc t	4527306		
	SN-1	gtt tat gta aag ta			
	SN-3	gta gtg gaa ttt ttt ttg gga agt t			
BCL2	Bi-BCL2-F1	ttg aag ttt ttt ttg att gtt ttg t	**NT_033903** 9342524	297	60
	Bi-BCL2-R1	taaatc aac cct ttc caa caa cta c	9342820		
	SN-1	tgt att ttt gtg tt			
CD44	Bi-CD44-F1	ata gtt ttt ttt gtt tgg gtg tgt t	**NT_009237** 33947855	254	
	Bi-CD44-R1	cca cca aaa ctt atc cat aat atc c	33948109		
	SN-2	agt ttt tgt tag gtt			
DAPK-1	Bi-DAPK-1-F1	taa aag gat tgg aga ttg atg tat ga	**NT_023935** 19278411	192	56
	Bi-DAPK-1-R1	taa tac ccc ctt ctt tac cta cca aat t	19278602		
	SN-3	tgg atg att att a			
RARB	Bi-RARb-F1	aga ggt agg agg gtt tat ttt ttg t	**NT_022517** 25409788	249	61
	Bi- RARb-R1	aat cat tta cca ttt tcc aaa ctt act	25410036		
	SN-1	ttg agg att ggg atg t			
	SN-2	agt agg gtt tgt ttg ggt at			
TNFRSF10C	Bi-TNFRSF10C-F1	gga tgt ttg gtt ttg gtt att tg	**NT_023666** 1335087	208	60
	Bi-TNFRSF10C-R1	tat aaa ttc cac ctt aac tcc ctc c	1335294		
	SN-1	gag tta ggg gaa gaa ttg ggt ttt			
